# Increased Regulatory T Cells Precede the Development of Bronchopulmonary Dysplasia in Preterm Infants

**DOI:** 10.3389/fimmu.2020.565257

**Published:** 2020-09-30

**Authors:** Julia Pagel, Nele Twisselmann, Tanja K. Rausch, Silvio Waschina, Annika Hartz, Magdalena Steinbeis, Jonathan Olbertz, Kathrin Nagel, Alena Steinmetz, Kirstin Faust, Martin Demmert, Wolfgang Göpel, Egbert Herting, Jan Rupp, Christoph Härtel

**Affiliations:** ^1^Department of Pediatrics, University of Lübeck, Lübeck, Germany; ^2^Department of Infectious Diseases and Microbiology, University of Lübeck, Lübeck, Germany; ^3^German Center for Infection Research (DZIF), Partner Site Hamburg-Lübeck-Borstel-Riems, Lübeck, Germany; ^4^Department of Pediatrics, University Hospital Hamburg-Eppendorf, Hamburg, Germany; ^5^Department of Medical Biometry and Statistics, University of Lübeck, Lübeck, Germany; ^6^Research Group Medical Systems Biology, Christian-Albrechts-University Kiel, Kiel, Germany; ^7^University Children's Hospital, University of Würzburg, Würzburg, Germany; ^8^PRIMAL (Priming Immunity at the Beginning of Life) Consortium, Lübeck, Germany

**Keywords:** regulatory T cells, Tregs, bronchopulmonary dysplasia, BPD, preterm infant, neonate, Foxp3

## Abstract

Regulatory T cells (Tregs) are important for the ontogenetic control of immune activation and tissue damage in preterm infants. However, the role of Tregs for the development of bronchopulmonary dysplasia (BPD) is yet unclear. The aim of our study was to characterize CD4+ CD25+ forkhead box protein 3 (FoxP3)+ Tregs in peripheral blood of well-phenotyped preterm infants (*n* = 382; 23 + 0 – 36 + 6 weeks of gestational age) with a focus on the first 28 days of life and the clinical endpoint BPD (supplemental oxygen for longer than 28 days of age). In a subgroup of preterm infants, we characterized the immunological phenotype of Tregs (*n* = 23). The suppressive function of Tregs on CD4+CD25- T cells was compared in preterm, term and adult blood. We observed that extreme prematurity was associated with increased Treg frequencies which peaked in the second week of life. Independent of gestational age, increased Treg frequencies were noted to precede the development of BPD. The phenotype of preterm infant Tregs largely differed from adult Tregs and displayed an overall naïve Treg population (CD45RA+/HLA-DR-/Helios+), especially in the first days of life. On day 7 of life, a more activated Treg phenotype pattern (CCR6+, HLA-DR+, and Ki-67+) was observed. Tregs of preterm neonates had a higher immunosuppressive capacity against CD4+CD25- T cells compared to the Treg compartment of term neonates and adults. In conclusion, our data suggest increased frequencies and functions of Tregs in preterm neonates which display a distinct phenotype with dynamic changes in the first weeks of life. Hence, the continued abundance of Tregs may contribute to sustained inflammation preceding the development of BPD. Functional analyses are needed in order to elucidate whether Tregs have potential as future target for diagnostics and therapeutics.

## Introduction

Bronchopulmonary dysplasia (BPD) is a major complication of premature birth and has been linked to chronic pulmonary inflammation leading to a high risk of mortality and long-term morbidities (1–5). Hence, there is an urgent need to develop early markers for the prevention of BPD.

We herein hypothesize that regulatory T cells (Treg) including their immunological phenotype and immunosuppressive capacities play a pivotal role for pathways of BPD development. This hypothesis is supported by several aspects. First, Tregs are crucial mediators of maintaining immune homeostasis. Second, Tregs are involved in a number of chronic, inflammation-mediated respiratory diseases. In line with this, alterations in Treg frequency and suppressive capacity are a risk factor for the susceptibility to infectious agents as well as the development of inflammatory, autoimmune, and allergic diseases in humans and animals (6–10). Third, immunoregulatory cell populations such as Tregs are a unique cell population for the complex mechanisms of the transition from intra- to extrauterine life. The immune functions of the semi-allogeneic fetus are adapted to prevent potentially damaging inflammation, which leads to temporary immunosuppression in the neonatal period. This ontogenetic specificity may enable colonization of the commensal microbiota to be tolerated after birth and avoids inflammatory tissue damage (11), however, it might predispose the neonate to infection. In term infants, many regulatory cell types including T helper type 2 (Th2) cells (12, 13), myeloid-derived suppressor cells (14), erythroid CD 71+ cells (15), regulatory B cells (16), and Treg (17–19) are involved in the control of inflammation. The neonatal period of the preterm infant is even more vulnerable and characterized by ineffective responses to pathogens, significant risk for sustained inflammation and intestinal dysbiosis (20–27). Forkhead box protein 3 (FoxP3)-expressing Tregs assume an indispensable role in regulating the immune response by performing a suppressor function through different mechanisms of action (28). Currently, complementary studies on the role of Tregs are seeking to uncover the therapeutic potential that these cells can offer for countless autoimmune and infectious diseases as well as for cancer (29–31). The pivotal role of Tregs for immunoregulation in preterm infants is displayed by a negative correlation of Treg frequency and gestational age, which has been shown in several studies (18, 19, 32–36). These studies have also shown a naïve Treg phenotype (CD45RO-/ CD45RA+) in cord blood of term and preterm infants, but comparable suppressive capacity of Tregs from term infants to adults after antigen exposure in *in vitro* assays (32, 34, 35, 37–40). Generally, effector Tregs are characterized by markers such as downregulation of CD45RA surface receptor and upregulation of the proliferation maker Ki67+, suppressive receptor CTLA4, activating marker HLA-DR, recruiting marker CCR6 as well as the surface enzyme CD39 leading to increasing inhibitory adenosine level (41). In addition, HELIOS+ Tregs are described to be important for their suppressive function (42). However, there is no study characterizing the effector Treg phenotype in preterm infants. In terms of clinical outcome, another study has demonstrated a fetal sheep model of chorioamnionitis, which leads to inflammatory changes in many organ systems after birth and a decrease in the number of Tregs in the fetal gut (43, 44). Decreased Treg percentages have also been described in human cord blood from infants with chorioamnionitis (45). Moreover, we described an increased Treg rate in preterm infants with early-onset sepsis (EOS) (46). In the context of BPD, CD4+ T cells along with Tregs have been shown to be reduced in cord blood (47). However, the development of Tregs during the first weeks of life of preterm infants and their role for the distinct susceptibility to BPD is yet unknown. Therefore, we conducted an observational longitudinal study to evaluate the Treg levels and phenotypes during the neonatal period of preterm infants and their association with clinical outcome parameters. Moreover, we tested the suppressive Treg capacity from preterm infants in comparison with term neonates and adults.

## Materials and Methods

### Study Cohort

We performed a single-center observational study in the Department of Pediatrics at the University Hospital of Lübeck and enrolled preterm infants as part of our IRoN (Immunoregulation of the Newborn) study project. Data were collected from infants born between 1 October 2014 and 30 September 2018. The inclusion criteria were as follows: preterm infants with gestational age 23+0 and below 37+0 weeks with need for in-hospital treatment, but without lethal abnormalities. In a subgroup of preterm infants, we analyzed the phenotype of the Tregs and used adult blood as controls. In addition, cord blood of preterm infants born below 37 weeks of gestation was collected to perform a Treg suppression assay. As control, cord blood of term infants born above 37 weeks of gestation and adult blood was used. After written informed consent was given by the parents or legal representatives, infants were enrolled by the attending physicians.

### Ethics

Written informed consent was obtained from parents or legal representatives on behalf of the infants enrolled into our study. The study parts were approved by the local committee on research in human subjects at the University of Lübeck (IRON AZ 15-304). All blood samples were obtained within a medically required blood withdrawal procedure. The additional blood volume obtained for research purposes (<1% of whole body blood volume per blood sampling) was in line with current guidelines of the European Medical Agency on the investigation of medicinal products in term and preterm infants; Committee for Medicinal Products for Human Use and Pediatric Committee (PDCO, 2006).

### Definitions

#### Gestational Age

Gestational age was calculated from the best obstetric estimate based on early prenatal ultrasound and obstetric examination. Small-for-gestational age (SGA) was defined as a birth weight less than the 10th percentile for gestational age according to gender-specific standards for birth weight by gestational age in Germany (48).

#### Early-Onset Sepsis (EOS)

EOS was defined as sepsis occurring within the first 72 h of life.

#### Late-Onset Sepsis (LOS)

LOS was defined as sepsis occurring after the first 72 h of life, but before 25 days of life.

#### Clinical Sepsis

Clinical sepsis was defined as the condition when neonatologists decided to treat the infant with antibiotics and continue for at least 5 days due to the following reasons: two clinical signs of systemic inflammatory response: temperature > 38°C or <36.5°C, tachycardia > 200/min, new-onset or increased frequency of bradycardias or apneas, hyperglycemia > 140 mg/dl, base excess < 210 mval/l, changed skin color and increased oxygen need; and one laboratory sign: C-reactive protein > 10 mg/l, platelet count < 100/nl, immature/total neutrophil ratio > 0.2 and white blood cell count < 5/nl (49, 50).

#### Blood Culture-Confirmed Sepsis

Blood culture-confirmed sepsis was defined as clinical sepsis with proof of causative agent in the blood culture.

#### Necrotizing Enterocolitis (NEC) and Focal Intestinal Perforation (FIP)

NEC and FIP were defined as need for surgery due to spontaneous intestinal perforation or necrotizing enterocolitis (Bell stage ≥2).

#### Bronchopulmonary Dysplasia (BPD)

BPD was defined as need for oxygen supplement for 28 days and longer.

#### Cause of Preterm Delivery

The cause of preterm delivery was determined at the discretion of the attending obstetrician, specifically: (1) preterm labor (labor refractory totocolytic agents) or suspected amniotic infection syndrome [AIS; labor ± rupture of membranes, increased maternal inflammatory markers without any other cause (CRP > 10 mg/l or elevation of white blood cell count > 16,000/ml)] or severe AIS [maternal fever (≥38.0°C), increased maternal inflammatory markers without any other cause (CRP > 10 mg/l or elevation of white blood cell count > 16,000/ml), labor ± rupture of membranes, fetal or maternal tachycardia, painful uterus and foul-smelling amniotic fluid]; (2) pre-eclampsia (pregnancy-induced maternal hypertension, edema, proteinuria); (3) pathological Doppler (e.g., Arteria umbilicalis Doppler, Ductus venosus flow, Arteria cerebri media Doppler) or intrauterine growth restriction as diagnosed by the attending specialist for antenatal ultrasound; and (4) others, including placental abruption, cholestasis, etc.

#### Antenatal Corticosteroids

Antenatal corticosteroids (betamethasone, intramuscular injection) were administered to women at risk for preterm birth between 24 and 34 weeks' gestation.

### Flow Cytometry of Tregs

The Treg percentage was determined via flow cytometry as previously described (44). Briefly, EDTA whole blood samples were stored at room temperature and processed within 24 h after withdrawal. Due to the limits of our study design (informed parental consent, preterm delivery on weekday, sufficient blood volume), we were not able to obtain blood samples for Treg quantification on days 1 to 28 of life of all infants. We included Treg data on day 1, 7, 14, 21, and 28 of life in the analyses. If data were not available for one of the scheduled days of blood withdrawal, we used data from venipunctures performed in the timeframe of ±3 days.

A cell viability test was performed to ensure cell viability after 24 h, which was over 95% (Fixable Viability Dye eFluor®780 conjugated, eBioscience, Thermo Fisher Scientific, Waltham, MA, USA). A whole blood staining with fluorochrome-labeled antibodies to characterize T cell populations was performed. For staining we used cell permeabilization and fixation reagents (FoxP3/Transcription Factor Staining Buffer Set; eBioscience, Thermo Fisher Scientific, Waltham, MA, USA). For multi-color flow cytometry analysis, cells were first stained with surface antibodies specific for CD3/fluorescein isothiocyanate (FITC) (clone OKT3; eBioscience, Thermo Fisher Scientific, Waltham, MA, USA), CD4/phycoerythrin (PE) (clone M-T466; MiltenyiBiotec, BergischGladbach, Germany), CD25/Pacific Blue, BV421 (clone BC96, BioLegend, San Diego, CA, USA) followed by intranuclear staining for FoxP3 (APC/eFluor660, clone PCH101; eBioscience, Thermo Fisher Scientific, Waltham, MA, USA). FoxP3 intranuclear staining was performed according to the manufacturer's protocol following cell surface staining. The fixed and stained cells were diluted in fluorescence activated cell sorter (FACS) staining buffer (eBioscience, Thermo Fisher Scientific, Waltham, MA, USA) and stored directly at 4°C. Flow cytometric analysis was performed within 4 days with a BD LSR II cytometer and analyzed with FACS Diva software (BD Bioscience, San Jose, CA, USA) and FlowJo (Tree Star, Ashland, OR, USA). Lymphocytes were determined by their position in the forward-/side-scatter plot (size/granularity) and co-expression of CD3/CD4/CD25/FoxP3 was necessary to identify the Treg cells within (44). In a subgroup of preterm infants and adults, we additionally stained the following markers on the surface: CD4/FITC (clone RPA-T4), CD8/Alexa 700 (clone RPA-T8), CD25/BV421 (clone BC96), HLA-DR/BV510 (clone L243), CD45RA/BV711 (clone HI100), CTLA-4(CD152)/PE/Dazzle 594 (clone BNI3), CD39/PE (clone A1), CCR6/PE-Cy7 (clone G034E3), CD127/BV785 (clone A019D5), as well as intracellular: Helios/PerCP-Cy5.5 (clone 22F6), Ki-67/BV605 (clone Ki67, BioLegend, San Diego, CA, USA), and FoxP3/eFluor660 (clone PCH101; eBioscience, Thermo Fisher Scientific, Waltham, MA, USA). The gating strategy for the phenotyping of Tregs is depicted in Supplementary Figure 1. Single antibody stainings were used to calculate the compensations and compensation beads for the large panel. Fluorescence minus one (FMO) controls were used to establish gating boundaries and to identify any background spread of fluorochromes (Supplementary Figures 3, 4).

### Treg Suppression Assay

EDTA cord blood samples were stored at room temperature and processed within 24 h after withdrawal. First, CD4^+^CD127^low^CD25^+^ Tregs were enriched using the complete kit for human CD4^+^CD127^low^CD25^+^ regulatory T cells (Stemcell Technologies, Vancouver, Canada) according to manufacturer's instructions. One to four milliliter of cord blood was used for the Treg isolation and purity was assessed using the flow cytometry Treg staining protocol mentioned above but adjusting the Foxp3 Fixation/Permeabilization working solution by an additional 1:4 dilution due to lower cell numbers. The Treg purity after isolation was 85–95% gated on living CD4^+^ singlet cells. Isolated Tregs were then activated for 3 days *in vitro*. Therefore, 1 to 5 × 10^4^ Tregs were seeded per round-bottom 96-well in 200 μl ImmunoCult™-XF T Cell Expansion Medium (Stemcell Technologies, Vancouver, Canada) supplemented with 5% autologous heat-inactivated serum, 500 U/ml interleukin (IL)-2 (eBioscience, Thermo Fisher Scientific, Waltham, MA, USA), 5 ng/ml transforming growth factor (TGF)β (eBioscience, Thermo Fisher Scientific, Waltham, MA, USA), and Penicillin (10000U)/Streptomycin (10 mg/ml) (P/S) (Sigma-Aldrich Corporation, St. Louis, USA). Additionally, Treg inspector beads (MiltenyiBiotec, BergischGladbach, Germany) were added in a 1:1 bead to cell ratio according to manufacturer's instructions for activation and proliferation of isolated Tregs.

After 3 days, CD4^+^ CD25^−^T cells were isolated from peripheral blood of a healthy adult donor using the EasySep™ Direct HumanCD4^+^ T Cell Isolation Kit followed by the EasySep™ Human CD25 Positive Selection Kit (Stemcell Technologies, Vancouver, Canada) which can be used for CD25 depletion according to manufacturer's instructions. To reduce the between donor variability of CD4+ CD25^−^ T cells, the same donor was used for CD4^+^ CD25^−^ T cells isolation in each experiment. Following isolation, CD4^+^ CD25^−^ T cells were labeled with carboxyfluoresceinsuccinimidyl ester (CFSE) to track the proliferation rate. Thus, 0.5 to 1 × 10^6^ CD4^+^ CD25^−^ T cells were incubated with 3 μM CFSE in 1,000 μl 0.1% Bovine serum albumin (BSA) in PBS buffer for 5 min at room temperature in the dark. To stop the reaction, CFSE-labeled CD4^+^ CD25^−^ T cells were washed twice by adding 10 ml ImmunoCult™-XF T Cell Expansion Medium supplemented with 10% heat-inactivated fetal bovine serum (FBS).

To assess the suppressive capacity of isolated Tregs, 1 × 1 0^4^ autologous CFSE-labeled CD4^+^ CD25^−^ T cells were seeded per round-bottom 96-well in a 1:1 and 2:1 co-culture with activated Tregs in 200 μl ImmunoCult™-XF T Cell Expansion Medium supplemented with 10% FBS, 125 U/ml IL-2, 2 ng/ml TGFβ, and P/S. Additionally, Treg inspector beads were added in a 1:1 bead to cell ratio according to manufacturer's instructions. As a positive proliferation control, CFSE-labeled CD4^+^ CD25^−^ T cells were seeded alone with the same suppression culture medium and ratio of Treg inspector beads. As negative controls, either CFSE-labeled CD4^+^ CD25^−^ T cells were seeded alone with the same suppression culture medium but without beads or unlabeled CD4^+^ CD25^−^ T cells were seeded alone with and without beads. All experiments were done in triplets. The co-culture was incubated for 4 days at 37°C in 5% CO_2_ in the dark.

After an overnight incubation, the positive and negative controls were stained with CD4/PE and a 1:2000 dilution of the fixable viability dye/eFluor®780 for 20 min at room temperature in the dark, washed and resuspended in 200 μl FACS buffer for flow cytometry analysis at the BD FACSCanto™ II cytometer to assess the CFSE staining. After 4 days of co-culture, all samples were stained with CD4/PE and fixable viability dye/eFluor®780 for 20 min at room temperature in the dark as described above. Proliferation of CD4^+^ CD25^−^T cells was determined of single, living CD4+ cells in the CFSE^+^ gate, which was determined using the negative controls. Single antibody stainings were used to calculate the compensations. Cell viability was always above 90%.

### Statistical Analysis

Linear Regression was used to analyze the influence of the gestational age at birth on the Treg frequency measured on the different merged days of life. Correlations were evaluated using Spearman's rank correlation coefficient. Generalized estimating equation (GEE) with Gaussian error and autoregressive [AR(1)] correlation matrix was used to determine the association between the repeated measure (up to four times) of Tregs assessed on different days of life and the interaction effects between the measuring time (timepoint) and either AIS, EOS, LOS, or BPD and the interaction effects between timepoint, LOS and BPD. Gestational age was used for adjustment. All variables were time-invariant except for Tregs and timepoint. In case of significance in the GEE model for one of the interactions between timepoint with EOS, LOS, or BPD the non-parametric Mann-Whitney U test was used in the subset of preterm infants who did not develop EOS, LOS or BPD. In each gestational age group (23–28, 29–32, 33–36) Treg frequencies between day 1–3 and 4–10, and day 4–10 and 11–17 were tested.

Additionally, in the subset of preterm infants born before 29 weeks of gestation the Treg frequencies of infants developing BPD were compared with infants not developing BPD measured on different days of life (1–3, 4–10, 11–17) by using the non-parametric Mann-Whitney U-test. This data analysis was performed using R studio version 3.5.2 (The R Foundation). Plots were generated using R package ggplot2 (3.2.1). The R-function geeglm [package geepack (1.2-1)] was used for the GEEs. The type I error level was set to 0.05. For the main analysis the type I error level was adjusted for multiple testing for 10 tests (for 5 linear regression and for 5 interactions in the GEE) using the method of Šidák-Holm (α_SH_) to control the familywise error rate. In the subset analysis of healthy preterm infants the type I error level of 0.05 was adjusted for multiple testing for two tests in each age group using the method of Šidák-Holm. The phenotyping of Tregs and the suppression assay were analyzed and visualized with GraphPad Prism 7. Differences between groups were assessed using ANOVA. The type I error level was set to 0.05. The method of Šidák-Holm was used for corrections for multiple testing.

## Results

### Criteria for Cohort Inclusion

We screened a large cohort of preterm infants (*n* = 527) between ≥23 ± 0 and <37± 0 weeks of gestation. We enrolled 382 preterm infants in our study cohort and prospectively collected peripheral blood samples during the first weeks of life. One hundred and forty five infants were excluded due to early death, lethal malformations or refused consent of parents or legal representatives. For the analysis of CD3+CD4+CD25+FoxP3+ Treg frequencies infants with longitudinal collection of blood samples (at least 1 sample in the first 28 days of life) were included. Detailed clinical data of the study cohort are described in Table 1. The preterm infants were divided into three gestational age groups: group 1: ≥23 + 0 – 28 + 6 weeks (*n* = 114), group 2: ≥29 + 0 – 32 + 6 (*n* = 152) and group 3: ≥33 + 0 – 36 + 6 (*n* = 116) weeks + days of gestational age.

**Table 1:** Clinical characteristics of the study cohort.

	**23–28 weeks *n* = 114**	**29–32 weeks *n* = 152**	**33–36 weeks *n* = 116**	**All preterm *n* = 382**
Gestational age(weeks)	26.6 ± 1.6	31 ± 1.1	34.1 ± 0.7	30.6 ± 3.1
Birth weight (g)	916 ± 272	1506 ± 308	2110 ± 471	1512 ± 584
SGA	13(11.4)	16 (10.6)	24 (20.9)	53 (13.9)
Gender (female)	47 (41.2)	91 (59.9)	59 (50.9)	197 (51.6)
Multiple	34 (30.1)	50 (33.3)	39 (33.9)	123 (32.5)
LOS	30 (26.3)	12 (7.9)	0 (0)	42 (11.0)
EOS	21 (18.4)	17 (11.2)	3 (2.6)	41 (10.7)
AIS	67 (60.4)	55 (37.2)	26 (23.4)	148(40.0)
NEC	5 (4.5)	2 (1.3)	0 (0)	7 (1.9)
FIP	8 (7.0)	0 (0)	0 (0)	8 (2.1)
BPD	57 (50.4)	6 (4.0)	0 (0)	63 (16.6)
**Mode of delivery**				
Spontaneous	7 (6.2)	14 (9.3)	36 (31.3)	57 (15.1)
Elective C/S	98 (87.5)	115 (76.7)	66 (57.4)	279 (74.0)
Emergency C/S	7 (6.2)	21 (14.0)	13 (11.3)	41 (10.9)
**Cause of delivery**				
Preterm labor or AIS	88 (78.6)	82 (55.0)	77 (67.5)	247 (65.9)
Pre-eclampsia	7 (6.2)	13 (8.7)	10 (8.8)	30 (8.0)
Pathological Doppler	20 (17.9)	60 (40.3)	28 (24.6)	108 (28.8)
Others	41 (36.6)	37 (24.8)	23 (20.2)	101 (26.9)

*SGA, small for gestational age (birth weight <10th percentile); C/S, cesarean section; AIS, amniotic infection syndrome; LOS, late-onset sepsis; EOS, early-onset sepsis; NEC, necrotizing enterocolitis; FIP, focal intestinal perforation; BPD, bronchopulmonary dysplasia (need for supplemental oxygen > 28 days). Data are described as n (%) or mean ± SD. Multiple causes of preterm birth were possible*.

### The More Premature the Higher Postnatal Treg Frequencies in the First Weeks of Life

The percentage of CD3+CD4+CD25+FoxP3+ Tregs correlated negatively with gestational age in preterm infants on days 1–3 (−0.25, *p* < 0.0001, adjusted type I error level α_SH_ = 0.0051; Supplementary Figure 2), 4–10 (−0.340, *p* < 0.0001, α_SH_ = 0.0057), 11–17 (−0.246, *p* = 0.0007, α_SH_ = 0.0064), and 18–24 (−0.130, *p* = 0.0353, α_SH_ = 0.0127) of life. However, Treg frequency and gestational age converged on day 28 of life (−0.07, *p* = 0.35, α_SH_ = 0.017) (Supplementary Table 1).

### Treg Frequencies of Preterm Infants Display a Peak in the Second Week of Life

**Figure 1:**
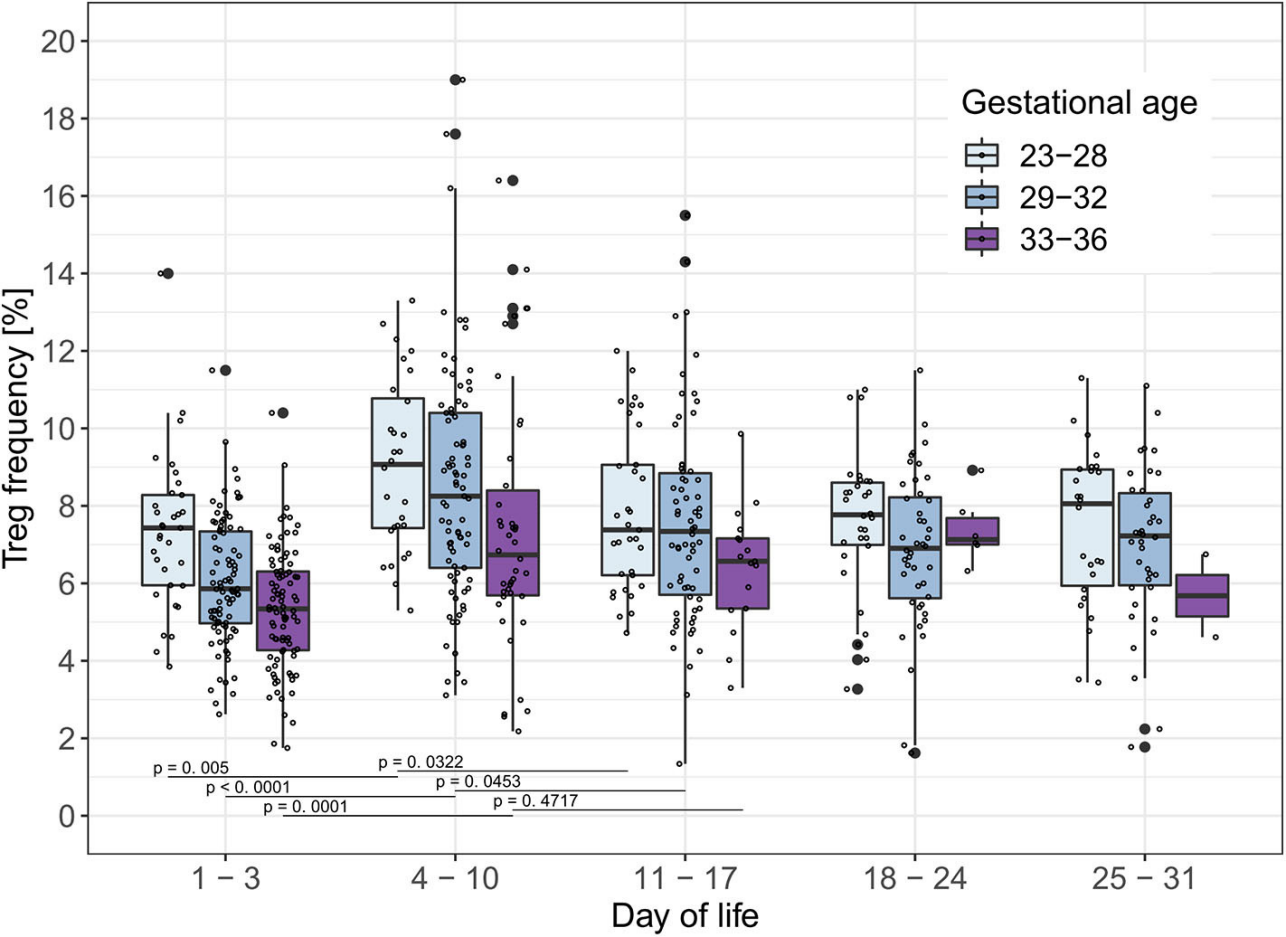
Treg frequencies [%] show a peak on day 4–10 of life in preterm infants without BPD, EOS or LOS. Treg frequencies [%] from preterm infants of three gestational age groups without EOS, LOS or BPD are displayed on different time points within the first month of life. Comparisons of Treg frequencies between days of life within each gestational age group showed an increase of the Treg frequencies in all gestational age groups from day 1–3 to 4–10 of life and a decrease in preterm infants <33 weeks of gestational age from day 4–10 to day 11–17 of life (Mann-Whitney U-test, type I error level corrected for two tests in each age group with Šidák-Holm (Day of life 1–3 to 4–10: α_SH_= 0.0253, 4–10 to 11–17: α_SH_= 0.05); GA 23–28: Day of life 1–3 to 4–10 (*p* = 0.005), 4–10 to 11–17 (*p* = 0.0322);GA 29–32: Day of life 1–3 to 4–10 (*p* < 0.0001), 4–10 to 11–17 (*p* = 0.0453); GA 33–36: Day of life 1–3 to 4–10 (*p* = 0.0001), 4–10 to 11–17 (*p* = 0.4717). Small dots represent value of one preterm infant.

To analyze the influence of time on Treg frequencies further, we visualized Treg frequencies over the course of the first 28 days of life once a week within each gestational age group excluding infants who developed EOS, LOS, or BPD (Figure 1). We observed a significant increase of Treg frequencies from day 1–3 of life to day 4–10 of life in all gestational age groups (23–28 weeks: *p* = 0.005; 29–32 weeks: *p* < 0.0001; 33–36 weeks: *p* = 0.0001; α_SH_ = 0.0253, respectively). This Treg peak on day 4–10 decreased again on day 11–17 of life, especially in the two smallest gestational age groups of preterm infants (23–28 weeks: *p* = 0.0322; 29–32 weeks: *p* = 0.0453, 33–36 weeks: *p* = 0.4717; α_SH_ = 0.05, respectively).

### Increased Treg Frequencies Precede the Development of BPD

**Figure 2:**
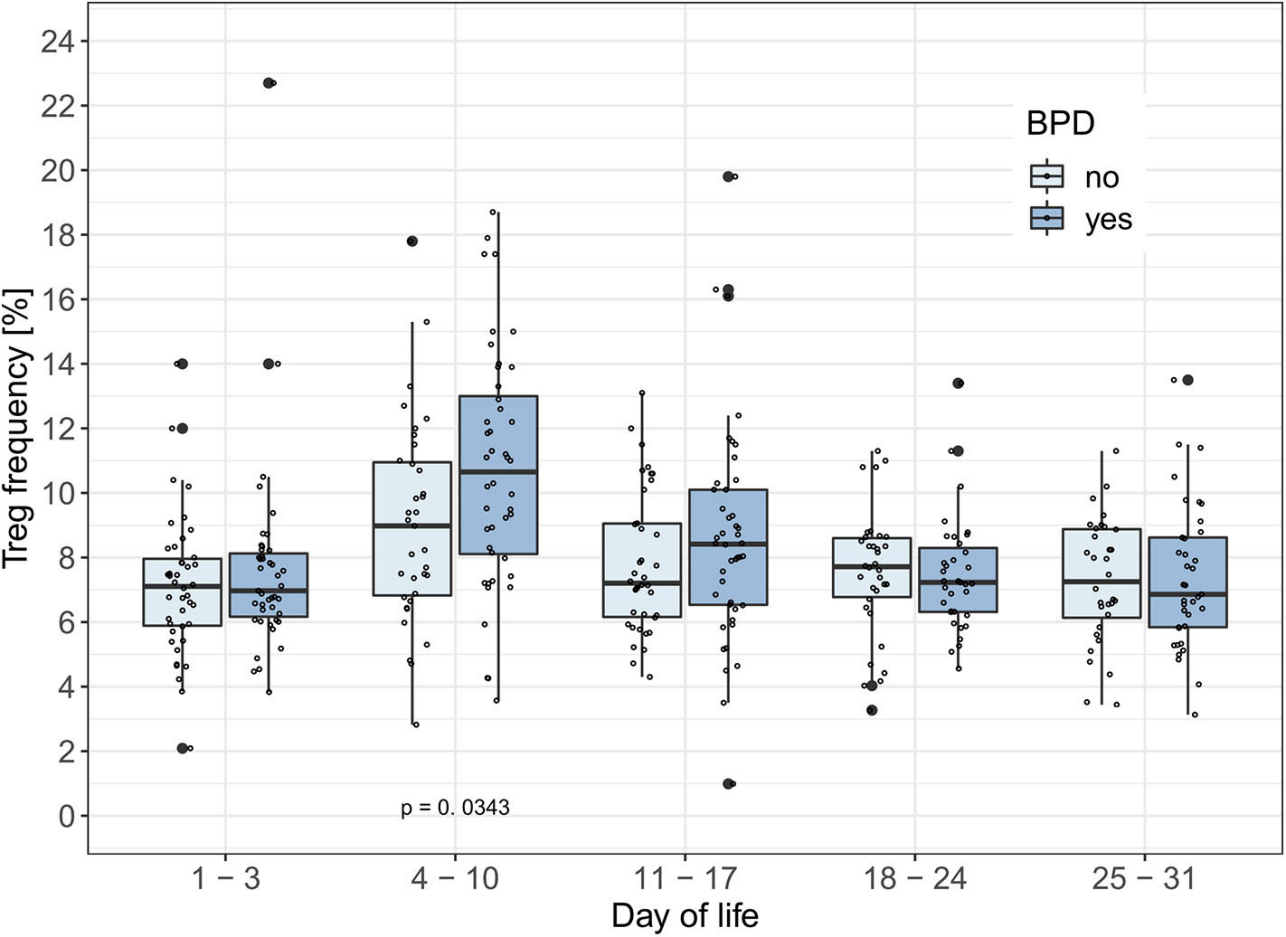
BPD patients have increased Treg frequencies between day 4–10 of life. Treg frequencies [%] are displayed over the first month of life in preterm infants developing bronchopulmonary dysplasia (BPD) within the smallest gestational age group of 23–28 weeks. The generalized estimating equation (GEE) analysis associated changes in Treg frequencies with the interaction between time point and BPD (Tab. 2: −0.657, *p* = 0.002, α_SH_ = 0.0073). Comparisons of Treg frequencies between BPD and non-BPD preterm infants showed significantly increased Treg rates in BPD patients on day of life 4–11 (Mann-Whitney U-test: Day of life 1–3: *p* = 0.5918; Day of life 4–10: *p* = 0.0343; Day of life 11–17: *p* = 0.2179). Small dots represent value of one preterm.

To test the hypothesis that Treg frequencies are associated with clinical outcome parameters, we applied the GEE statistical model. BPD over time (−0.657, *p* = 0.002, adjusted type I error level α_SH_ = 0.0073) and the interaction of BPD, LOS and postnatal timepoint (1.175, *p* = 0.0025, α_SH_ = 0.0085) were associated with the dynamics of Treg frequencies. LOS over time (−0.428, *p* = 0.0229, α_SH_ = 0.0102) was not significantly associated with the dynamics of Treg frequencies after adjustment for multiple testing. In addition, the interactions postnatal timepoint and EOS or AIS had no significant impact on the Treg frequencies in this model (Table 2). To confirm our observation of increased Treg frequencies preceding the development of BPD, we additionally compared Treg frequencies in preterm infants <29 weeks of gestation with BPD (*n* = 57) and without BPD (*n* = 56). Preterm infants subsequently developing BPD showed increased Treg frequencies before disease onset on day 4–10 (*p* = 0.0343) in comparison with infants without BPD (Figure 2). At later time points, no change in Treg frequencies was visible until day 70 of life (data not shown). The rate of antenatal administration of corticosteroids to the mothers in the BPD vs. non-BPD group (within the group <29 weeks of gestational age) was 92% vs. 87% (*p* = 0.5041).

**Table 2:** Results of the generalized estimating equation (GEE) models to determine the association between Treg frequencies on different postnatal timepoints with clinical parameters.

	**Estimate**	**SE**	**95% CI**		***p*-value**	**Adjusted type I error level**
Gestational age	−0.277	0.054	−0.383	−0.172	<0.0001	
Timepoint	0.313	0.093	0.131	0.495	0.001	
LOS	0.475	0.796	−1.086	2.036	0.551	
BPD	1.953	0.788	0.408	3.498	0.013	
EOS	0.684	0.869	−1.020	2.388	0.431	
AIS	−0.395	0.370	−1.121	0.331	0.287	
**Interactions**						
Timepoint × LOS	−0.428	0.188	−0.797	−0.059	0.023	**0.0102**
Timepoint × BPD	−0.657	0.212	−1.073	−0.241	0.002	**0.0073**
Timepoint × EOS	−0.135	0.243	−0.611	0.341	0.579	**0.050**
Timepoint × AIS	−0.085	0.131	−0.341	0.171	0.517	**0.0253**
LOS × BPD	−2.986	1.253	−5.440	−0.531	0.017	
Timepoint × LOS × BPD	1.175	0.389	0.413	1.938	0.003	**0.0025**

*Gestational age was included as confounder. Interaction effects of timepoint and AIS, timepoint and EOS, timepoint and LOS, timepoint and BPD, as well as timepoint and LOS and BPD, were tested (AIS, amniotic infection syndrome; EOS, early-onset sepsis; LOS, late-onset sepsis; BPD, bronchopulmonary dysplasia (need for supplemental oxygen > 28 days); type I error level was adjusted for 5 of 10 tests with Šidák-Holm; CI, confidence interval; SE, standard error).*.

### Distinct Phenotype of Tregs in Preterm Infants

**Figure 3:**
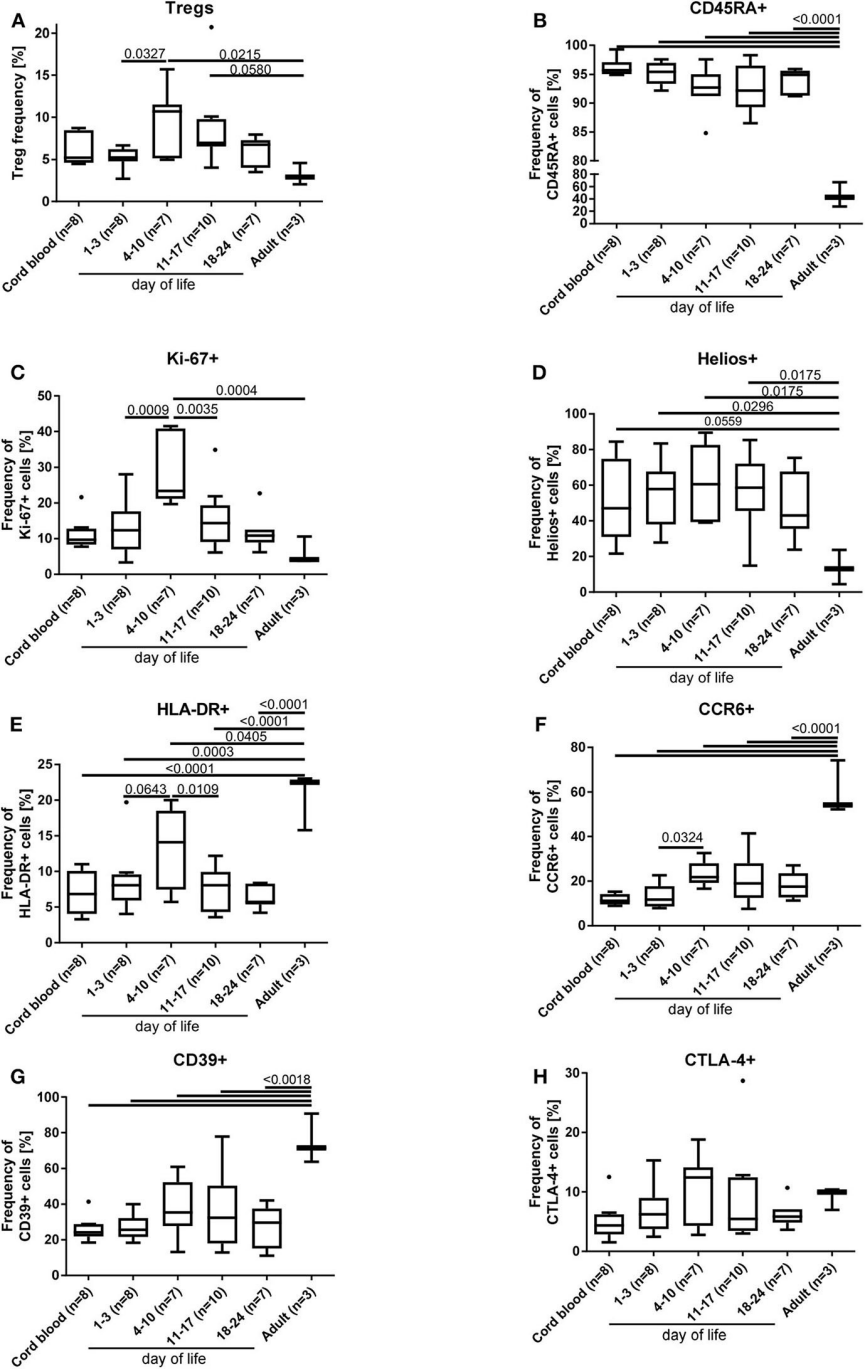
Phenotyping of Tregs **(A)** from preterm infants revealed an increased proliferation [Ki67+**(C)**] and activation [HLA-DR+ **(E)** and CCR6+ **(F)**] on day 4–10 of life, when the Treg peak was also detected under physiological conditions. In addition, naïve CD45RA+ **(B)**, proliferating Ki67+ **(C)** and HELIOS+ **(D)** Tregs of preterm infants were significantly increased compared to adult Tregs, whereas the activation marker expression of HLA-DR **(E)**, CCR6 **(F)**, and CD39 **(G)** was reduced on preterm Tregs. CTLA-4 **(H)** expression on Tregs did not show differences. The gating strategy is depicted in Figure 1 (*n* = 23, ANOVA followed by Šidák-Holm, *p*-values are presented in the figure).

In a subgroup of 23 preterm infants (mean gestational age of 29+5 weeks), phenotyping of the Tregs was performed including the markers HLA-DR, CD45RA, CTLA-4, CD39, CCR6, Helios, and Ki-67 and compared to adult blood samples (*n* = 3). The phenotype of Tregs from preterm infants is different to the adult Treg phenotype and displays an overall naïve Treg population (CD45RA+/Helios+) with an upregulation of the proliferation marker Ki67 (Figures 3B–D), whereas the activation marker HLA-DR, the migration and recruitment marker CCR6 and the surface bound ectonucleotidase CD39 were downregulated (Figures 3E–G), especially in the first days of life. CTLA-4 expression showed no differences (Figure 3H). In parallel with the Treg peak described above, we observed an increase of the proliferation marker expression Ki67, of the activation marker HLA-DR, and of the migration and recruitment marker CCR6 on day 4–10 of life (Figures 3A,C,E,F).

### Suppressive Capacity of Tregs Is Increased in Preterm Infants

**Figure 4:**
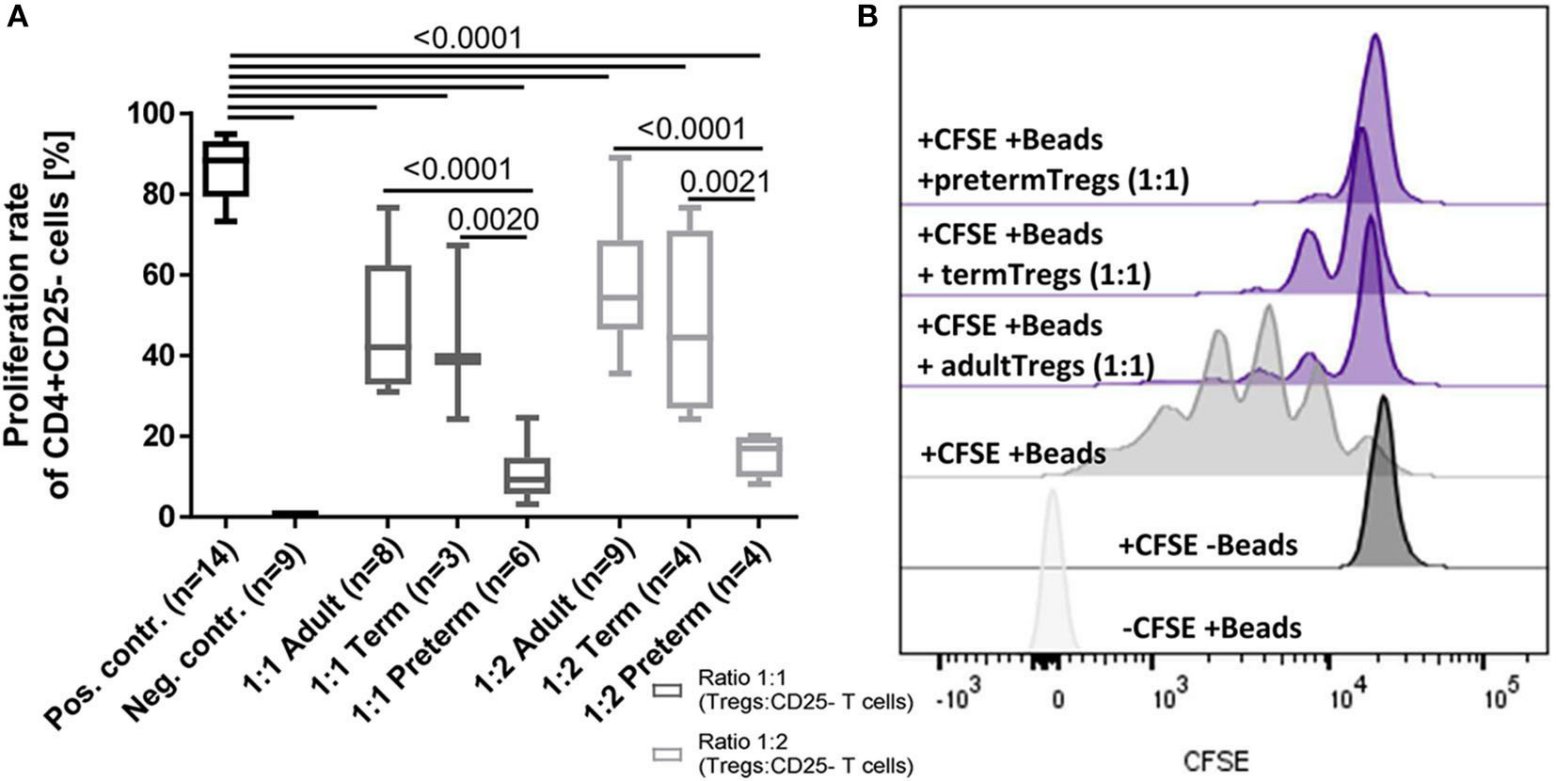
Suppressive capacity of regulatory T cells (Tregs) derived from preterm infants on the proliferation of CD4+ CD25- T cells was significantly stronger after 4 days of co-culture compared to Tregs from term infants and adults. **(A)** Proliferation rate of CD4+ CD25– T cells after 4 days of co-culture with Tregs from adults, term and preterm infants in a 1:1 or 2:1 ratio. The positive control was generated without Tregs. Proliferation was determined via CFSE signal using flow cytometry. **(B)** Representative stagged histograms show the negative staining control (-CFSE) with beads, the negative proliferation control (+CFSE) without beads, and the positive proliferation control (+CFSE, +Beads) without suppressive Tregs (ANOVA followed by Šidák-Holm, *p*-values are presented in the figure).

To investigate functional differences of Tregs, the suppressive capacity of Tregs isolated from six cord blood samples of preterm infants (mean gestational age 34+2 weeks) was compared to four cord blood samples of term infants (mean gestational age 38+4 weeks) and adult blood samples (*n* = 9). In this subgroup, no inflammatory or other diseases were reported during the duration of their hospital stay and all babies were born via cesarean section. Preterm Tregs had a significantly stronger suppressive capacity compared to term infants and adults (Figure 4). The proliferation rate of CD4+ CD25– T cells was reduced by preterm Tregs from 88% (median, pos. control) to 9% (median, 1:1 CD4+CD25– T cells : preterm Tregs), whereas the proliferation was only reduced to 42% by adult Tregs and to 39% by term infant Tregs (median, 1:1 cell ratio). The suppressive capacity was also shown to be dependent on the number of Tregs in the co-culture, since a 2:1 ratio of CD4+CD25– T cells to Tregs led to higher proliferation rates compared to a 1:1 cell ratio within the different groups of preterm, term and adult derived Tregs (Figure 4).

## Discussion

In this single-center prospective longitudinal study we demonstrated that increased Treg frequencies in the first 2 weeks of life precede the development of BPD in preterm infants. Our observation was independent of important confounders such as gestational age and sepsis. The increased Treg frequencies are accompanied by dynamic changes in the phenotype of the Treg populations in preterm infants. After birth a naïve Treg population (CD45RA+/Helios+) is dominating while a more activated Treg phenotype pattern (CCR6+, HLA-DR+, Ki-67+) develops in the second week of life accompanied by a peak in the Treg frequencies. Accordingly, a higher immunosuppressive capacity of Tregs from preterm neonates against CD4+CD25– T cells was noted as compared to adults.

BPD is regarded as a lung phenotype derived from a multifactorial set of risks and exposures, e.g., immaturity, structural damage due to ventilation and oxygen requirement, inflammation or dysbalanced immune responses and metabolic deficits. There is consensus that preventive strategies of BPD, e.g., the stabilization of immune responses, need to target a critical window of postnatal development. There is a paucity of immunological studies performed with peripheral whole blood of those infants being susceptible for BPD. We therefore performed a longitudinal study of preterm infants during the vulnerable time frame of the first 4 weeks of life. Our study enabled us to investigate the influence of the Treg frequency including Treg phenotypes and function on clinical outcomes of the preterm infants.

As Tregs are crucial mediators for immune tolerance and inhibition of potentially harmful pro-inflammatory immune responses (7, 8, 10), our findings have implications for the understanding of the immune regulation of preterm infants. The negative correlation of Treg frequency and gestational age within the first 21 days of life (Supplementary Table 1 and Supplementary Figure 2) underlines the potential role of fetal Tregs to promote self-tolerance and tolerance to maternal antigens (17, 26, 51). During pregnancy the infiltration of maternal immune cells in the fetal blood and fetal lymphoid tissues induces the development of fetal Tregs to maintain maternal–fetal tolerance (51, 52) and therefore explains the high percentages of Tregs in early preterm compared to full term neonates (19, 32, 33, 35, 36, 46). The convergence of Treg frequencies on day 28 of life (Supplementary Table 1) is in line with a systems-level analysis study of immune cells in peripheral blood of preterm infants compared to term infants, which described a convergence over the first month of life for the immune system but did not include Tregs in their analysis (53).

After birth, the neonate is exposed to a variety of antigens including the bacteria colonizing the skin and mucosae. Within this complex setting, our data suggest a unique role of Tregs for immunoregulation in the first weeks of life (Figure 1). Higher Treg frequencies in term infants at birth compared to adults shown by several studies (33, 35, 36, 46) suggest a physiological role in mediating tolerance during the first weeks of life enabling colonization and immune education to unknown antigens without a harmful inflammatory response. Herein, we show a more activated Treg phenotype around day 7 in preterm infants with an upregulation of the proliferation marker Ki67+, the activation marker HLA-DR and the recruiting marker CCR6 together with a peak of Treg frequencies (Figures 1, 3). This suggests an important time window which might be decisive for the immune-microbiota co-development.

To analyze the higher Treg frequencies on a functional level, we investigated phenotype and suppressive capacity of preterm Tregs. The overall naïve CD45RA+ Treg phenotype in preterm infants expressing lower levels of activating marker HLA-DR, recruiting marker CCR6, as well as suppressive surface enzyme CD39 at the beginning of life as compared to adults (32, 33, 35, 36, 46) suggests slightly less effective tolerance in preterm infants after birth (Figure 3). However, we detected a higher frequency of HELIOS+ preterm Tregs as well as a higher immunosuppressive capacity of preterm Tregs against CD4+/CD25– T cell proliferation as compared to term neonates and adults at birth (Figure 4). The higher suppressive capacity indicates a unique role of Tregs for preterm infants which might be induced by the phenotypic marker HELIOS as described by other studies (54, 55). Nevertheless, another study performed with human cord blood described that Tregs from preterm infants have less suppressive capacity on the proliferation of CD8–/CD25– T cells than those from term neonates and adults (45), while activation patterns were not different (34). These contradicting results are probably due to methodological differences such as cryopreservation and different time points of blood tests.

In our large-scale cohort, we noted increased Treg frequencies during the first 2 weeks of life preceding the development of BPD (Figure 2). This finding was independent of potential confounders such as gestational age or administration of antenatal corticosteroids to the mothers. This is in contrast to the observation of reduced Treg frequencies in cord blood of infants who developed BPD in a previous study (47). We selected peripheral blood as primary biomaterial, as cord blood might not reflect the immune cell frequencies in the first weeks of life and may be contaminated with maternal cells. In addition, Treg plasticity in the first weeks of life has to be taken into account. In other inflammatory lung diseases such as asthma previous studies have shown diverse results with Treg frequencies being up or downregulated which leads to the hypothesis that the role of Tregs might depend on the time point during disease progression (56, 57). Our cohort of preterm infants gives the unique opportunity to be able to analyze Treg frequencies before disease onset. Functional analyses are needed to elucidate the role of increased Treg frequencies and their phenotype before the onset of BPD. These studies should also address the research question if higher Treg numbers might increase the potential of polarization from a “suppressive” Treg phenotype to an “inflammatory” Th17 like phenotype, which is described in preterm infants with AIS (58). The plasticity of T cells is already well-recognized (59) and Th17 cells seem to play a role in other immune-mediated diseases such as asthma (60).

Thus, we propose early Treg dynamics as a potential target to predict or prevent BPD, especially for the most vulnerable preterm infants born before 29 weeks of gestation. Animal models are needed to reveal functional relevance of our findings. Our study can contribute to the future aim to develop an individualized diagnostic and treatment protocol for each preterm infant including immune therapeutic agents to ameliorate the prognosis.

Our study showed no difference in the Treg levels in patients with AIS compared to non-AIS infants in the first weeks of life. This is in contrast with previous studies, which used cord blood instead of peripheral blood (45). In fetal AIS sheep models, it was shown that the Treg frequencies in the fetal gut, thymus and lymph nodes are decreased due to intra-amniotic IL-1a expression and that inflammatory changes are spread out in many organ systems after birth (43, 44, 59–61). However, we did not confirm increased Treg frequencies in EOS and LOS patients (46), which might be due to the small number of affected babies.

The particular strengths of our study are the large sample size, the use of peripheral blood samples in a longitudinal fashion, the standardized observation of all infants under controlled NICU conditions and the detailed phenotyping of infants. We accounted for incomplete data sets (due to limited sample volume and pre-analytical time restrictions) with the generalized estimating equation model which adjusts the results for input parameters. Other potential limitations are the single-center observation. In addition, we used a relatively broad definition for BPD in our cohort. There is an ongoing discussion about the optimal definition of BPD and further studies are needed to reveal whether Tregs might predict the severity of respiratory and neurodevelopmental outcomes in preterm infants. Moreover, we used a CD4 CD25 FoxP3 staining approach for the Treg identification, which may include contamination by effector T cells. There is an ongoing controversial debate about the characterization of Tregs with different gating strategies and alternative gating strategies include CD4+ CD25+ CD127 low/–, CD4 +FoxP3 +CD127low/–, or even CD4 +CD25 +FoxP3 + CD127low/– Tregs. We therefore included CD127 staining in our large Treg panel and could confirm that the CD4 +CD25 +FoxP3 + cell population is mostly CD127low/– (Supplementary Figure 1). Finally, our study is hypothesis-generating, a functional relationship of both Tregs and effector T cells has yet to be examined in infants affected with BPD, respectively, compared to non-affected children. Tregs have been described to exert immunosuppression on the proliferation of naive T cells and the effector functions of differentiated T cells, B cells, NK cells, macrophages and dendritic cells (62). In line with this, future studies are needed to evaluate the effects of Tregs on the development of BPD in preterm infants, including the interaction of neonatal Tregs with other immunoregulatory cell types of innate and adaptive immunity.

Future *in vivo* studies could further reveal the question whether modulation of Tregs in preterm infants, either boosting or blocking regulatory responses, exert a new therapeutic option. In addition, further studies should evaluate distinct immunological profiles including Tregs as a diagnostic biomarker for individualized strategies to prevent inflammatory diseases such as BPD.

## Data Availability Statement

All datasets generated for this study are included in the article/Supplementary Material.

## Ethics Statement

The studies involving human participants were reviewed and approved by Local committee on research in human subjects at the University of Lübeck (IRON AZ 15-304). Written informed consent to participate in this study was provided by the participants' legal guardian/next of kin.

## Author Contributions

JP, CH, NT, and JR: experimental concept and design. JP, NT, AH, MS, JO, KN, AS, KF, and MD: performance/realization of experiments. JP, JR, WG, and EH: contribution of reagents, materials, and analysis tools. JP, NT, CH, TR, SW, and JR: data analysis. JP, NT, and CH: writing of paper. All authors contributed to the article and approved the submitted version.

## Conflict of Interest

The authors declare that the research was conducted in the absence of any commercial or financial relationships that could be construed as a potential conflict of interest.
