# Supplementary material for: Increased Regulatory T Cells Precede the Development of Bronchopulmonary Dysplasia in Preterm Infants
Source: Front Immunol. 2020 Sep 30;11:565257. doi: 10.3389/fimmu.2020.565257 (PMC7554370; doi:10.3389/fimmu.2020.565257)
Supplement: Supplementary file 1 [file Data_Sheet_1.pdf]

Supplementary Material

1 Supplementary Figures and Tables

1.1 Supplementary Tables

Supplemental table 1: Treg frequencies negatively correlate with gestational age until 21 days of life and converge at 28 days of life (linear correlation with gestational age; r_s: Spearman´srankcorrelation coefficient; type I error level was adjusted for 5 of 10 tests with Šidák-Holm).

Day of life	n	Estimate (p value)	Adjusted type I error level	r_s
1-3	284	-0.250 (< 0.0001)	0.0051	-0.378
4-10	231	-0.340 (< 0.0001)	0.0057	-0.336
11-17	179	-0.246 (0.0007)	0.0064	-0.267
18-24	139	-0.13 (0.0353)	0.0127	-0.158
25-31	119	-0.074 (0.3498)	0.0170	-0.037

1.2 Supplementary Figures

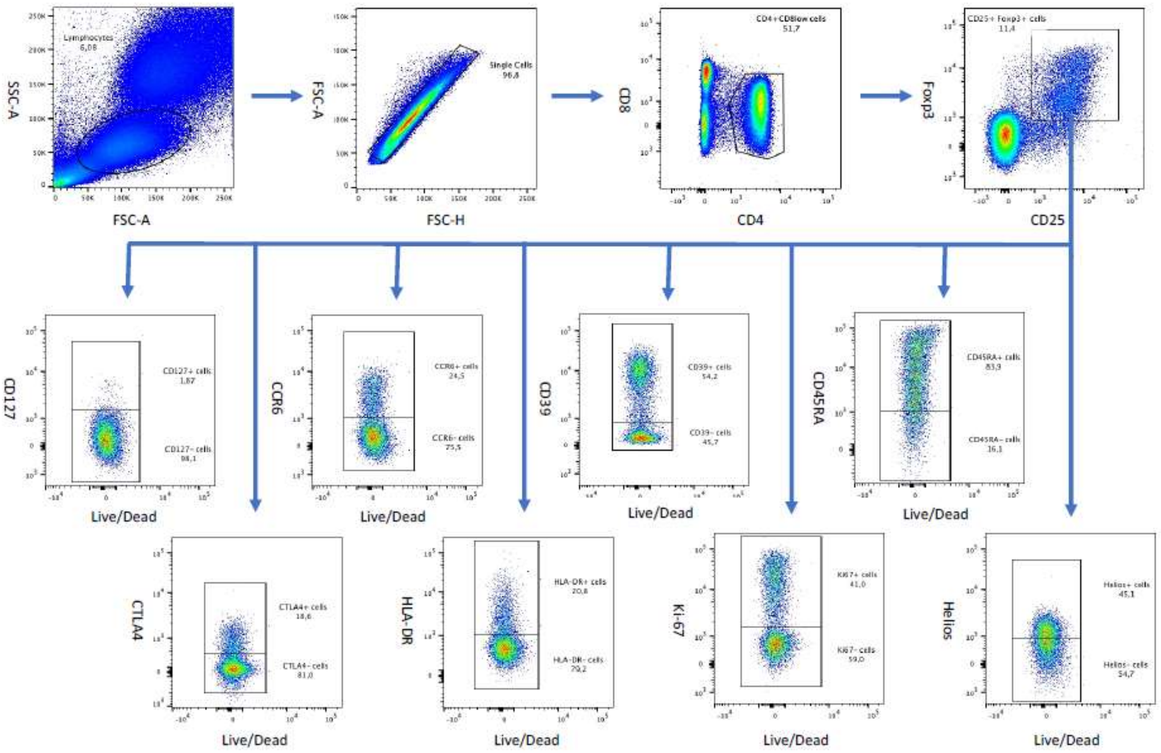

Supplemental figure 1: Gating strategy for Treg phenotyping. Representative gating in preterm infants from day 8 of life. We gated the following markers as percentage of CD4+ CD25+ and FoxP3+ regulatory T cells: alive and HLA-DR+, CD45RA+, CTLA-4+, CD39+, CCR6+, Helios+, and Ki-67+ cells. Single antibody stainings were used to calculate the compensations. Fluorescence minus one (FMO) controls were used to establish gating boundaries and to identify any background spread of fluorochromes.

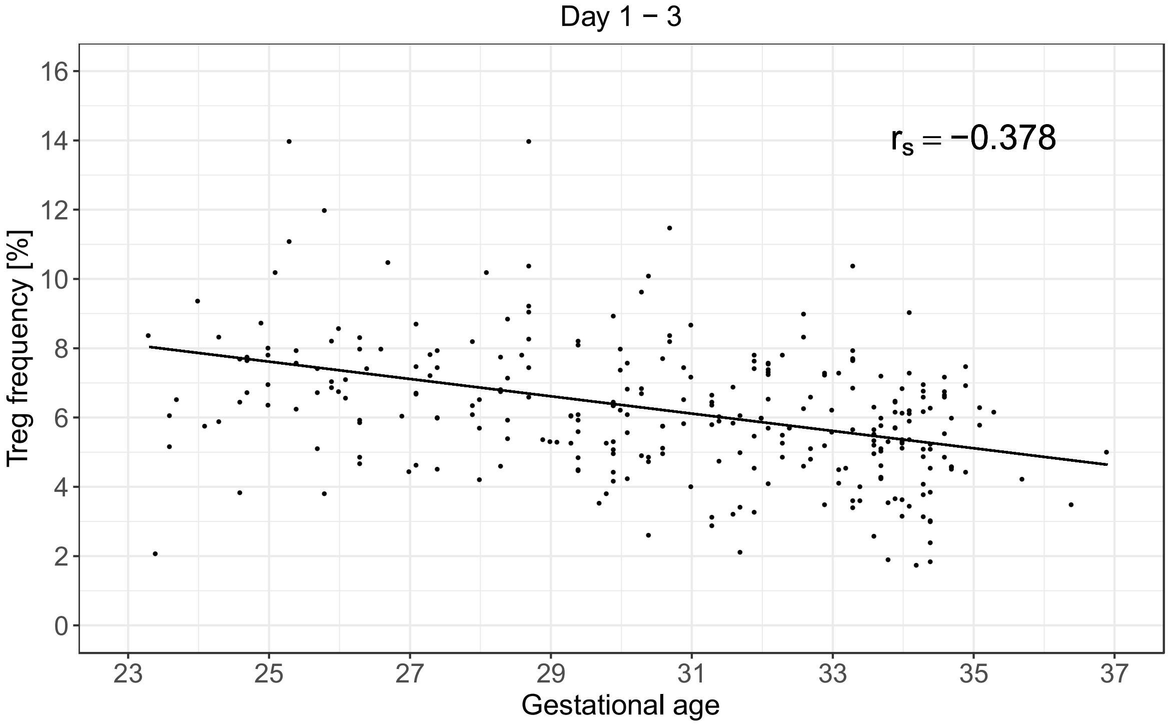

Supplemental figure 2: Treg frequencies negatively correlate with gestational age on day of life 1 to 3 (-0.25, p <0.0001; r_s: Spearman´s rank correlation coefficient).

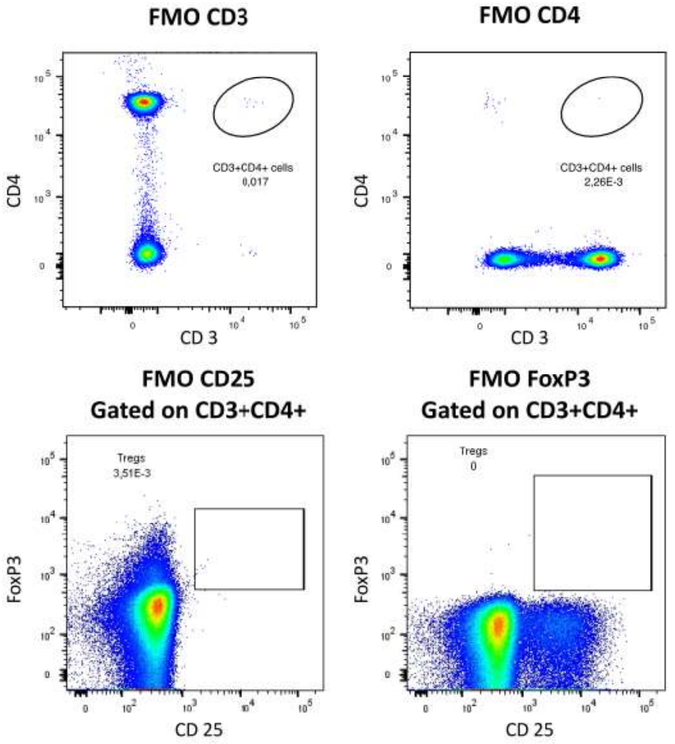

Supplemental figure 3: Fluorescence Minus One (FMO) Controls for Treg panel. Representative gating of FMO controls in preterm infants from day 3 of life. We gated the percentage of CD3+ CD4+ CD25+ and FoxP3+ regulatory T cells and used these controls to establish gating boundaries and to identify any background spread of fluorochromes.

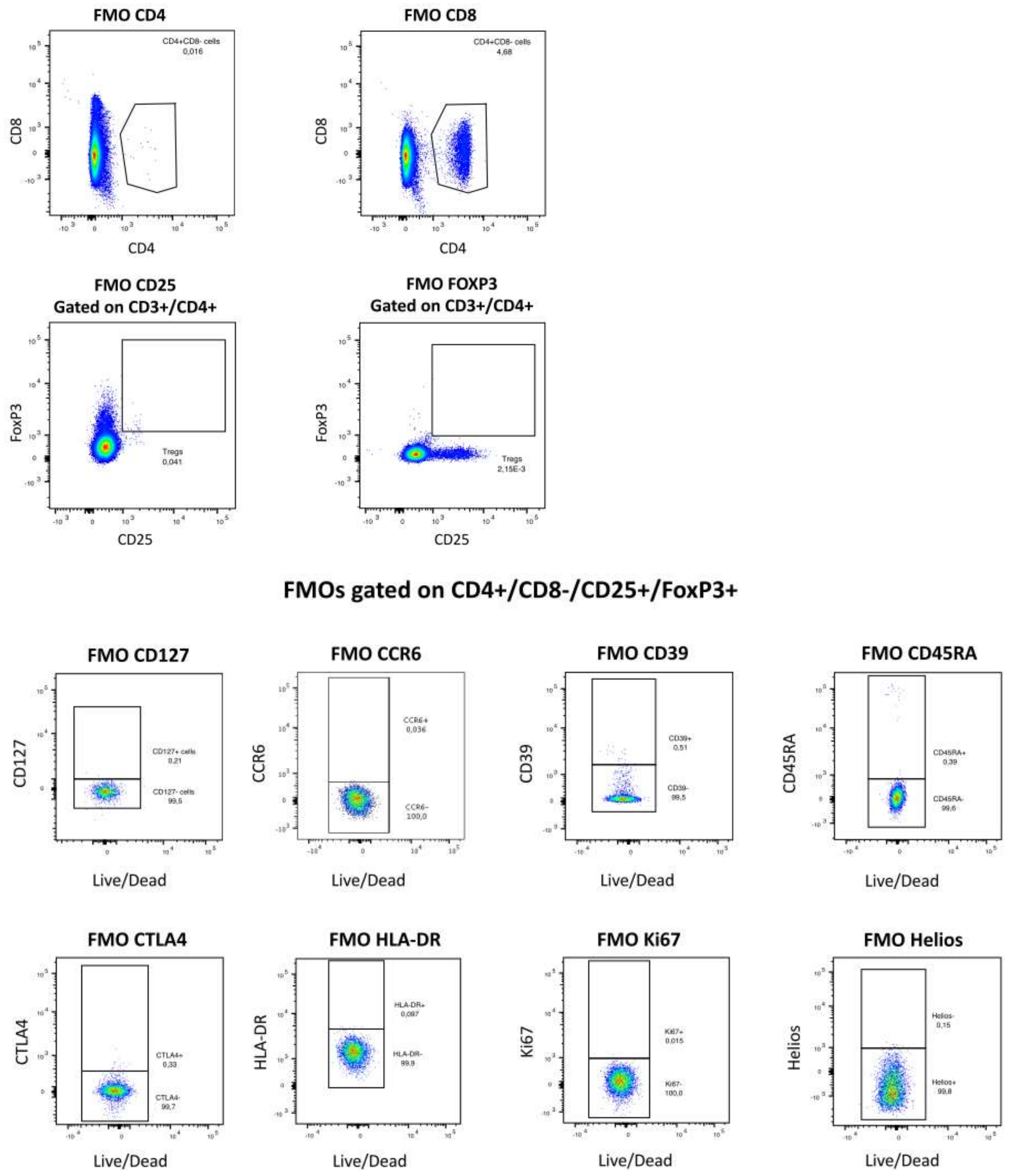

Supplemental figure 4: Fluorescence minus one (FMO) controls for Treg phenotyping panel. Representative gating of FMO controls in preterm infants from day 3 of life. We gated the following markers as percentage of CD4+ CD25+ and FoxP3+ regulatory T cells: HLA-DR+, CD45RA+, CTLA-4+, CD39+, CCR6+, Helios+, and Ki-67+ cells and used these controls to establish gating boundaries and to identify any background spread of fluorochromes.
